# The role of stimulus periodicity on spinal cord stimulation-induced artificial sensations in rodents

**DOI:** 10.1088/1741-2552/ad2b89

**Published:** 2024-03-05

**Authors:** Jacob C Slack, Sidnee L Zeiser, Amol P Yadav

**Affiliations:** 1 Joint Department of Biomedical Engineering, University of North Carolina at Chapel Hill and North Carolina State University, Chapel Hill, NC, United States of America; 2 Department of Biomedical Engineering, Purdue University Indianapolis, Indianapolis, IN, United States of America; 3 Department of Neurosurgery, UNC School of Medicine, Chapel Hill, NC, United States of America; 4 Neuroscience Center, University of North Carolina at Chapel Hill, Chapel Hill, NC, United States of America

**Keywords:** artificial sensations, spinal cord stimulation, neuroprosthesis, sensory feedback

## Abstract

*Objective.* Sensory feedback is critical for effectively controlling brain-machine interfaces and neuroprosthetic devices. Spinal cord stimulation (SCS) is proposed as a technique to induce artificial sensory perceptions in rodents, monkeys, and humans. However, to realize the full potential of SCS as a sensory neuroprosthetic technology, a better understanding of the effect of SCS pulse train parameter changes on sensory detection and discrimination thresholds is necessary. *Approach.* Here we investigated whether stimulation periodicity impacts rats’ ability to detect and discriminate SCS-induced perceptions at different frequencies. *Main results.* By varying the coefficient of variation (CV) of interstimulus pulse interval, we showed that at lower frequencies, rats could detect highly aperiodic SCS pulse trains at lower amplitudes (i.e. decreased detection thresholds). Furthermore, rats learned to discriminate stimuli with subtle differences in periodicity, and the just-noticeable differences from a highly aperiodic stimulus were smaller than those from a periodic stimulus. *Significance.* These results demonstrate that the temporal structure of an SCS pulse train is an integral parameter for modulating sensory feedback in neuroprosthetic applications.

## 1. Introduction

Neuroprosthetic devices and brain-machine interfaces (BMIs) have successfully demonstrated restoration of motor function in individuals suffering from impairments caused by neurological injuries and disorders [1–4]. However, methods that aim to restore the critical sensory functions of touch and proprioception via a non-visual pathway such as intracortical stimulation of the somatosensory cortex, deep brain stimulation, and stimulation of peripheral nerves are still under investigation [5–8]. An alternative method, spinal cord stimulation (SCS)—traditionally used for the treatment of chronic pain [9, 10], was recently shown to successfully generate artificial sensations in rats, monkeys, and human subjects [11–13]. Yet, the optimal spinal stimulation parameters that can reproducibly evoke naturalistic sensory perceptions are still to be determined.

The stimulation patterns used in traditional SCS systems comprise of tonic pulse trains with constant parameters of current amplitude, frequency, and pulse width. Recent studies have shown that variations in these principal parameters introduced by innovative paradigms such as burst-SCS, intensity modulated-SCS, and differential target multiplexed-SCS can improve the efficacy of the therapy being delivered [14–16]. Thus, there is strong evidence to suggest that alternative and innovative pulse parameters that deviate from traditional SCS need to be investigated for the purpose of inducing sensory perceptions. Since traditional pulse trains consist of fixed frequency, the pulses delivered are periodic in nature. But the naturalistic pattern of neural activity representing tactile and proprioceptive signals is not always periodic, and critical information about texture or stimulus location is often encoded in the precise temporal spiking pattern of cortical neurons [17, 18]. It is therefore argued that to mimic naturalistic sensations, it might be necessary to apply stochastic rate modulation by delivering pulse trains with a degree of aperiodicity or randomness [19, 20].

Previously, it was shown that SCS-induced sensory detection thresholds decreased with increasing frequency, pulse width, and duration of stimulation [12]. Moreover, although rats learned to discriminate SCS frequencies, and frequency discrimination obeyed Weber’s law, the stimulation train used was always periodic in nature. Thus, it needs to be investigated whether rats can learn to detect and discriminate aperiodic SCS pulse trains and whether varying periodicity changes detection and discrimination thresholds. This will allow the determination of upper and lower limits for periodicity as novel temporal stimulation patterns are explored. In this study, we used a two-alternative forced choice (2AFC) task to determine sensory detection thresholds at different levels of aperiodicity determined by the coefficient of variation (CV) of the interstimulus pulse interval. We also determined the just-noticeable difference (JND) in aperiodicity that rodents can successfully discriminate at multiple stimulation frequencies.

## 2. Methods

All animal procedures were approved by the Indiana University Institutional Animal Care and Use Committee and were performed in accordance with National Institute of Health Guide for the Care and Use of Laboratory Animals. Seven Long Evans rats (250–400 g) participated in the experiments (supplementary table 1).

### 2.1. Pre-training and spinal implant surgery

**Figure 1.**
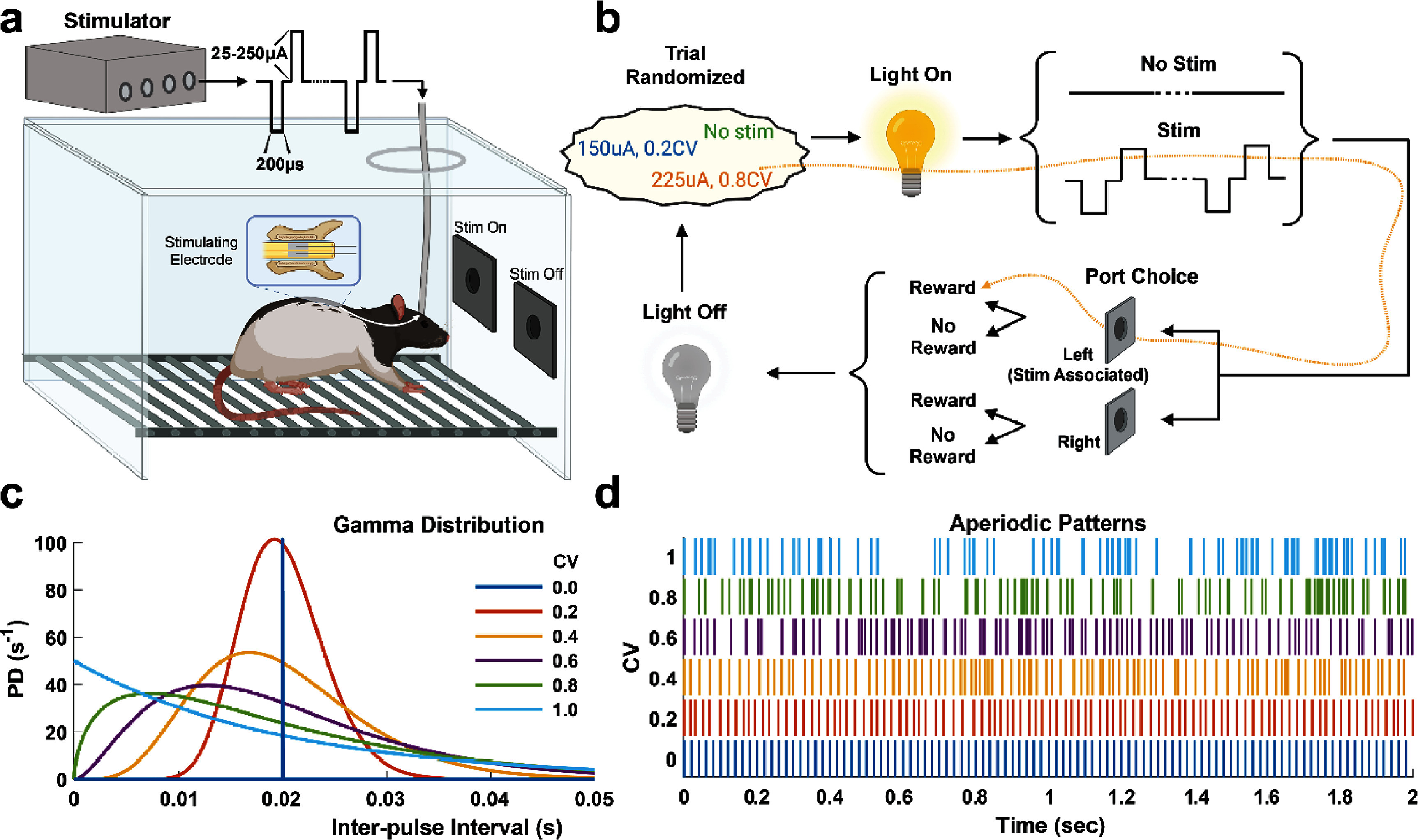
Behavioral task design and aperiodic SCS pulse-train detection learning. (a) Behavioral chamber with two water reward ports covered by doors for conducting detection and discrimination experiments. A microstimulator delivered biphasic, bipolar, charge-balanced pulse trains across the two-lead epidural electrode in freely moving rats. (b) Task flow of a detection trial. A session normally consisted of 200–400 trials. ‘Stim’ and ‘No Stim’ trials were randomly selected followed by a light indicator followed by presentation of a sensory cue for two seconds. Rats received either SCS for ‘Stim’ trials or absence of SCS for ‘No Stim’ trials. Reward port doors would then open allowing the rat to select either left or right port. A water reward paired with a short auditory cue was delivered if a rat chose the left port during a ‘Stim’ trial or the right port during a ‘No Stim’ trial. If an incorrect port was chosen, no reward was delivered paired with a long auditory cue. (c) Probability density function based on a gamma distribution to generate aperiodic template patterns using the coefficient of variation (CV) of the interstimulus pulse interval to vary the level of periodicity (CV = 0: periodic; CV = 1: highly aperiodic). (d) Resulting pulse train template patterns generated by the gamma distribution in ‘c’ for each CV value.

Upon animal delivery, baseline weights of all rats were taken, and rats were acclimated to human handling for 1–2 days. After acclimation, rats were moderately water-deprived and were placed in the operant training chamber for approximately one week to become comfortable with the experimental environment. The chamber contained two water dispensing reward ports on each side that were enclosed by doors and placed behind infrared (IR) beam sensors (figure 1(a)). A water reward was delivered when the rat completed a nose poke into the port, interrupting the IR beam. A light in the chamber would turn on to signify the start of each trial. Initially, the rats were rewarded with water after making a nose poke and licking at either spout. Following that, rats were trained to make nose pokes in each spout on alternate trials. The initial learning period took between 7–23 days, i.e. a consistent performance (>90%) on alternating reward port trials.

Once rats successfully learned how to interact with the operant chamber, custom two-contact SCS platinum electrodes (1 × 0.5 mm) spaced 0.25 mm and 0.025 mm thick with PFA coated stainless steel leads (A-M Systems, Sequim, WA, USA) were implanted epidurally underneath the T3–T5 vertebra using previously established procedures [12, 21–23]. To ensure minimal migration, the electrodes were also fixed to the vertebral process. The leads were then routed to an Omnetics connector (Omnetics Connector Corporation, MN, USA) and fixed to the skull with skull-screws (W. W. Grainger Inc., IL, USA) and dental acrylic (Colten Holding, Switzerland). After recovery, electrodes were tested using a custom biphasic/bipolar microstimulator [24]. Tests involved applying pulse-trains using stimulation parameters consistent with the experimental task (i.e, 50 Hz pulse-train above sensory threshold but below motor threshold).

### 2.2. Aperiodic SCS pulse-trains

SCS pulse-trains were bipolar, biphasic, and charge-balanced with 200 *μ*s phase duration and inter-phase interval of 50 *μ*s. To generate aperiodic SCS pulse-trains, inter-pulse interval values were pulled from a gamma distribution in MATLAB (MathWorks, MA, USA) using equation (1) [20].

\begin{equation*}f\left( {x{\text{|}}k,\theta } \right) = \frac{1}{{{\theta ^k}\Gamma \left( k \right)}}{x^{k - 1}}{e^{\frac{{ - x}}{\theta }}}\end{equation*}

with probability density function *f*, inter-pulse interval *x* in milliseconds, the gamma function Γ, shape parameter *k* and scale parameter θ. The degree of aperiodicity was modulated by changing the shape and scale parameters which are defined as follows:

\begin{equation*}k = {\raise0.7ex\hbox{$1$} \!\mathord{\left/ \right.} \!\lower0.7ex\hbox{${{\text{C}}{{\text{V}}^2}}$}}\end{equation*}

\begin{equation*}\theta = \mu {\text{C}}{{\text{V}}^2}\end{equation*}

where CV is the coefficient of variation and *μ* the mean inter-pulse interval. Larger CV values shift the peak of the probability density function leftward producing increasingly aperiodic patterns (figures 1(c) and (d)). For psychometric detection, six CV values were chosen ranging from 0 (periodic) to 1 incremented by 0.2. In discrimination experiments, ten CV values were used incremented by 0.1 from 0 to 1. In both cases, a template pattern was generated from equation (1) for each CV value at each frequency and used for all rats. The template pattern was limited to a two second window and contained equal pulse across CV values within a given frequency. For example, the template patterns for 50 Hz at 0.2 CV and 50 Hz at 1 CV both contained 100 pulses within the two-second stim period.

### 2.3. Behavioral task design

To learn the stimulation detection task, rats were trained on a 2AFC task. We used a behavioral chamber (Med Associates Inc., VT, USA) described above and previously [11, 12] but modified the electrical interface so that it could be custom-controlled by Arduino (Arduino LLC, Italy) and interfaced with MATLAB via a graphical user interface (GUI) (figure 1(a)). A session normally consisted of 200–400 trials. During a trial, the house light turned on followed by stimulation present (‘Stim’ trial) or stimulation absent (‘No Stim’ trial) (figure 1(b)). Trial selection was randomized programmatically. ‘Stim’ and ‘No Stim’ trials were randomly selected followed by a light indicator followed by presentation of a sensory cue for two seconds. Rats received either SCS for ‘Stim’ trials or absence of SCS for ‘No Stim’ trials. Reward port doors would then open allowing the rat to select either left or right port. A water reward paired with a short auditory cue was delivered if a rat chose the left port during a ‘Stim’ trial or the right port during a ‘No Stim’ trial. If an incorrect port was chosen, no reward was delivered paired with a long auditory cue.

### 2.4. Sensory detection task and psychophysics

For initial detection training, stimulation amplitude above sensory threshold but below motor threshold was determined before each session by the experimenter. Amplitude was incrementally increased until subtle twitching was observed around the implant location then reduced until twitching was no longer visible. Previously we used periodic pulse trains at 100 Hz for detection training, but here, the pulse-train (cathode leading with 200 *μ*s pulse width, 50 *μ*s interphase delay, 2 s duration) was delivered at 0.8 CV at 50 Hz. Once rats reached a correct detection rate of 80% (supplementary figure 1(a)), CV (0-1) and amplitude were randomized for ‘Stim’ trials at a given frequency (figure 1(d)). Amplitude ranges were selected for each frequency such that approximately 3–4 amplitudes were below and above sensory threshold and that maximum amplitude did not elicit significant twitching or discomfort (supplementary table 2). When 20 trials for all CV and amplitude combinations were completed, a different frequency was selected, and the process was repeated. Sensory detection thresholds were calculated as 75% of correct detections from sigmoid fits.

### 2.5. Initial sensory discrimination task

The sensory discrimination task also utilized a 2AFC task setup and training chamber. Prior to the session, each individual rat’s sensory threshold amplitude was determined with a 2 s periodic (0 CV) pulse train. This amplitude was constant throughout the session unless the rat was performing poorly and needed an adjustment. The stimuli varied with either a high (1) or low (0) CV. Other parameters such as pulse width (200 *μ*s), amplitude (determined and set at the beginning of each session), frequency (20, 50, 100, or 200 Hz), duration (2 s) remained the same throughout the session. For the initial discrimination task, the high CV was associated with the left reward port while the low CV corresponded to the right. Once each rat consistently showed the ability to discriminate between 1 CV and 0 CV by achieving a correct response rate >80%, they were advanced to the next task to determine JNDs in CV.

### 2.6. JND and sensory discrimination

After learning the initial CV discrimination (0 vs. 1 CV), the left port was held at a constant 1 CV (standard CV) while the right port CV (comparison CV) was randomized from 0 to 0.9 in increments of 0.1. The same experiment was then repeated while keeping the right port at a constant 0 CV (standard CV) and randomizing the left port CV (comparison CV) from 0.1 to 1. The standard CV was not changed during a particular session. Trial type and comparison CV were randomized by a programmatic algorithm. Discrimination performance was calculated as the fraction of total correct trials for each standard and comparison pair presentation. The fractions were plotted against their corresponding standard/comparison CV difference. A sigmoidal curve was then fitted to the points. The JND of CV was determined as the 75% correct percentage mark on the resulting curve. Once JND was determined for a particular frequency, the same procedures (initial discrimination training followed by CV randomization) were repeated at different frequency values to study additional trends.

### 2.7. Statistical analysis

**Figure 2.**
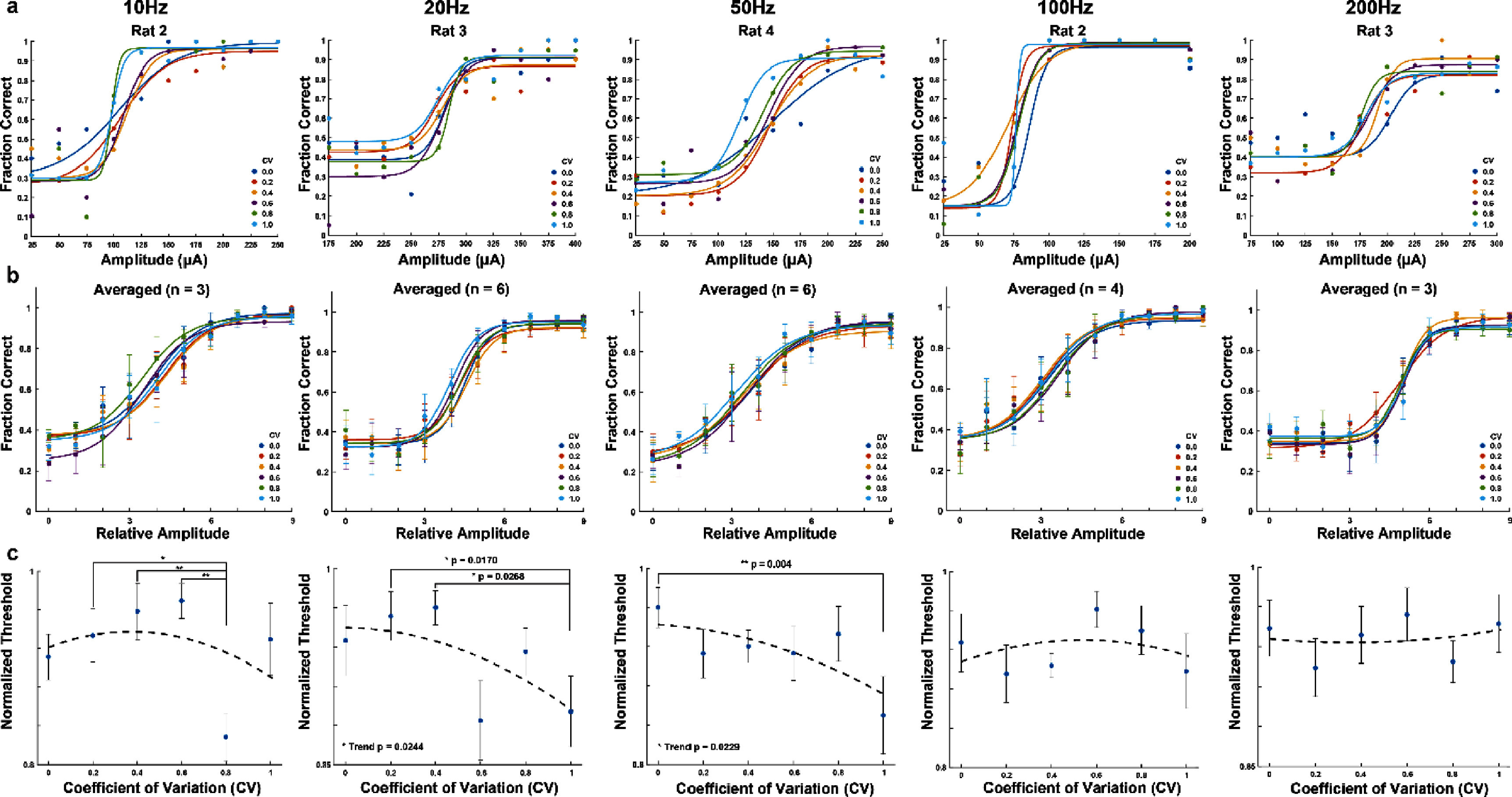
Psychometric analysis of SCS sensory detection for various degrees of aperiodicity at constant frequency. Columns indicate the frequency of SCS pulse-trains. (a) Representative psychometric detection curves for individual rats at multiple CV values at constant frequency. (b) Psychometric detection curves averaged across rats for each frequency (10, 20, 50, 100, 200 Hz; *n* = 3, 6, 6, 4, 3). *X*-axis depicts amplitude relative to individual rats (amplitude ranges subtracted by minimum amplitude and divided by step size). Circles and error bars indicate mean $ \pm \,$standard error. Traces for ‘*a*’ and ‘*b*’ are sigmoid fits for individual CV values across amplitudes. (c) Normalized detection thresholds across CV for constant frequency. For each rat, thresholds were determined from individual sigmoid fits as seen in ‘*a*’ as the 75% fraction of correctly detected stimuli and normalized by maximum threshold. Circles and error bars indicate mean $ \pm \,$standard error. *P*-values were calculated from repeated measures one-way ANOVA post-test for trend and multiple comparisons.

**Figure 3.**
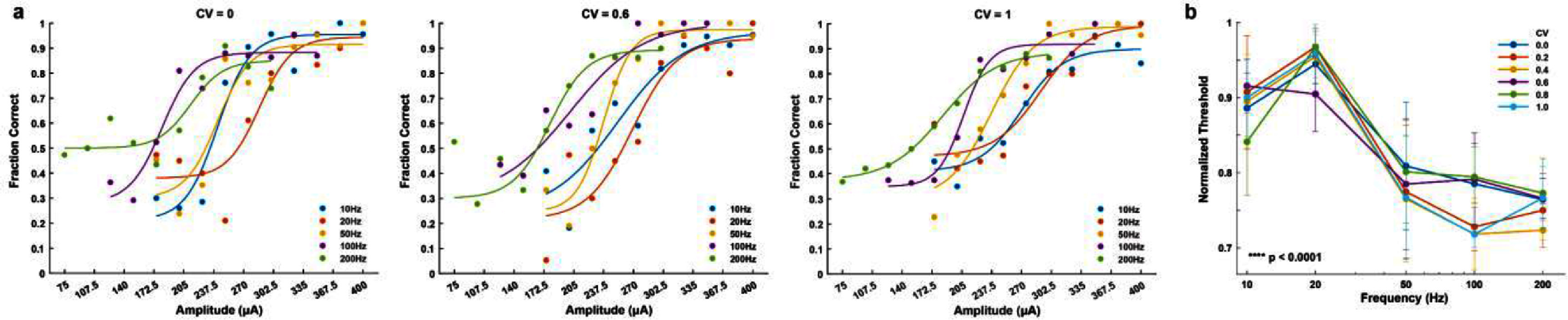
Psychometric analysis of sensory detection across frequency. (a) Representative psychometric detection curves across frequency for rat 3. Traces are sigmoid fits for multiple frequencies at a given CV value. (b) Normalized detection thresholds across frequency. Traces indicate normalized thresholds for a CV value at each frequency (10 Hz: *n* = 4 rats; 20 Hz: *n* = 6 rats; 50 Hz: *n* = 6 rats; 100 Hz: *n* = 4 rats; 200 Hz: *n* = 4 rats). Circles and error bars indicate mean $ \pm \,$standard error. *P*-value was calculated by one-way ANOVA.

Before performing statistical analysis, for figure 2(c)—thresholds for each CV within a given frequency were normalized by the maximum threshold for each rat, and for figure 3(b)—thresholds for each frequency within a given CV were normalized by the maximum threshold for each rat. Repeated Measures one-way ANOVA and one-way ANOVA with post-test for trend and multiple comparisons were applied to determine significant differences between thresholds at various degrees of periodicity and frequency respectively. A paired t-test was used to determine significant differences between JNDs at CV = 0 and CV = 1 (figure 5(e)).

## 3. Results

### 3.1. Sensory detection of aperiodic SCS pulse-trains

During initial training, six rats learned to detect cathode-leading, biphasic, aperiodic pulse-trains (frequency: 50 Hz, CV: 0.8, pulse-width: 200 ms, inter-phase delay: 50 *μ*s, amplitude: 190.8$ \pm $90.1 *μ*A, supplementary figure 1(a)) over the course of 11 $ \pm $5 sessions. To determine sensory detection thresholds at different CV values (0–1), amplitude and CV were randomized at each stimulation frequency (10–200 Hz), and psychometric curves were fit to the resultant detection performance values (figure 2(a)). Averaged detection thresholds across all rats showed that detection thresholds were significantly different between 0 CV and 1 CV stimuli at 50 Hz (figure 2(c), third column, *p* < 0.01, repeated measures (RM) one-way ANOVA multiple comparisons). Detection thresholds were also found to have a decreasing linear trend for both 20 Hz (figure 2(c), second column, *p* < 0.05, RM one-way ANOVA test for trend) and 50 Hz (figure 2(c), third column, *p* < 0.05, RM one-way ANOVA test for trend).

Analyzing detection performance across multiple frequencies demonstrated that detection thresholds decreased with increasing frequency for all CV values (figures 3(a) and (b), *p* < 0.0001, one-way ANOVA post-test for trend, and supplementary figure 2). Previously, we showed that detection thresholds decreased with increasing frequency [12], however, in that case, the stimulus pulse train was only delivered in a periodic form (corresponding to 0 CV here). Thus, our results establish that the relationship between detection thresholds and frequency is maintained independently of stimulus periodicity.

### 3.2. Sensory discrimination of aperiodic SCS pulse-trains

Following the detection behavioral task, five rats were trained to discriminate between SCS patterns that varied in CV while the amplitude, frequency, pulse-width, and duration were kept constant throughout a session. All five rats learned the initial task of discriminating between a CV of 1 and CV of 0; rat 1 and 4 successfully learned at 50 Hz while rats 2, 3, and 5 successfully learned at 20 Hz (supplementary figure 3). The rats achieved a consistent correct percentage rate ⩾80% between 8–34 d (supplementary figure 1(b)).

**Figure 4.**
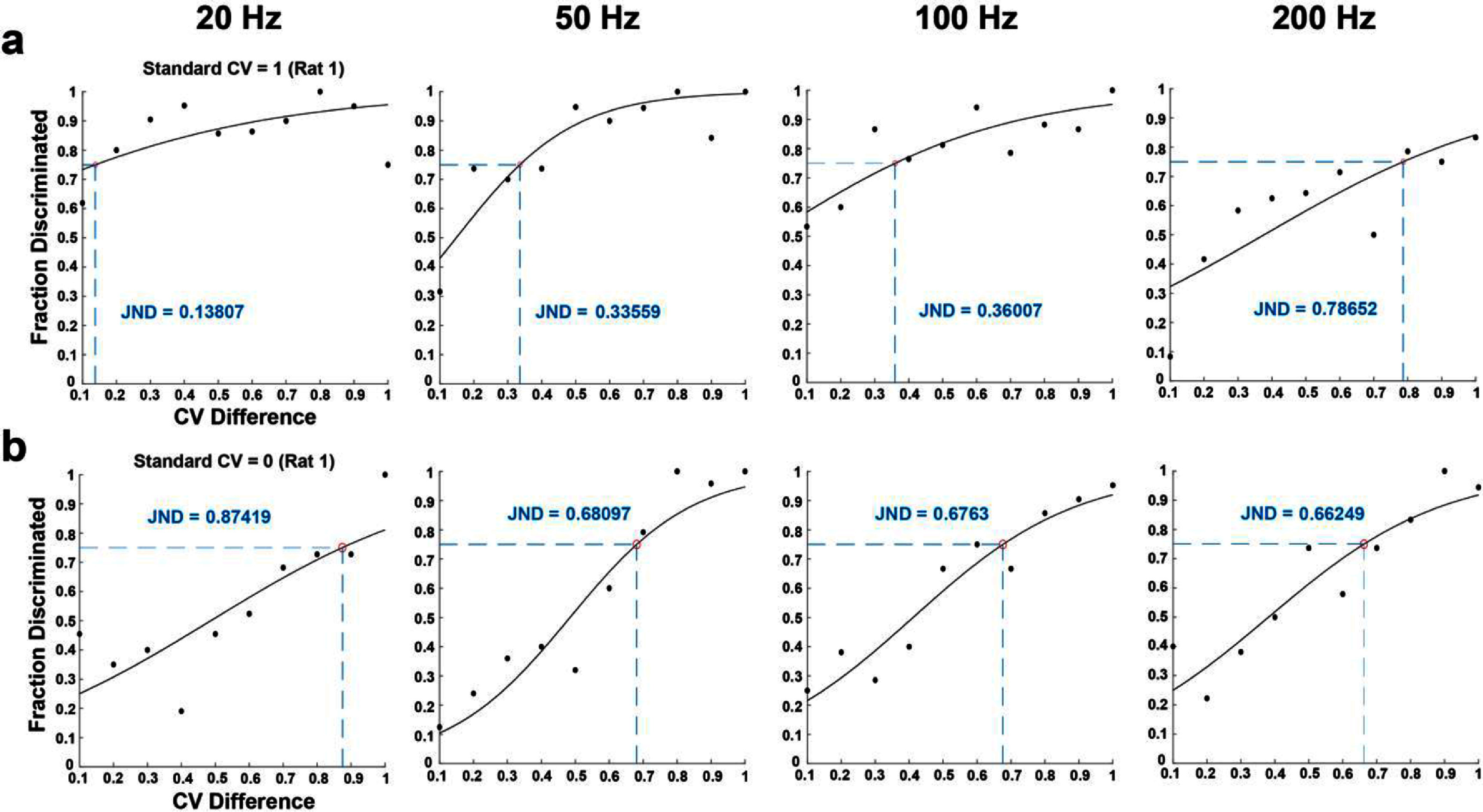
Psychometric analysis of sensory discrimination to determine just noticeable differences (JNDs) for a representative rat (rat 1). JND was taken as the CV difference value at the 75% correct mark on each sigmoidal curve. (a) Fraction of trials correctly discriminated from a standard CV of 1. (b) Fraction of trials correctly discriminated from a standard CV of 0. The circles in panels a and b indicate the fraction of correctly discriminated trials for each CV difference from the standard CV for that session.

**Figure 5.**
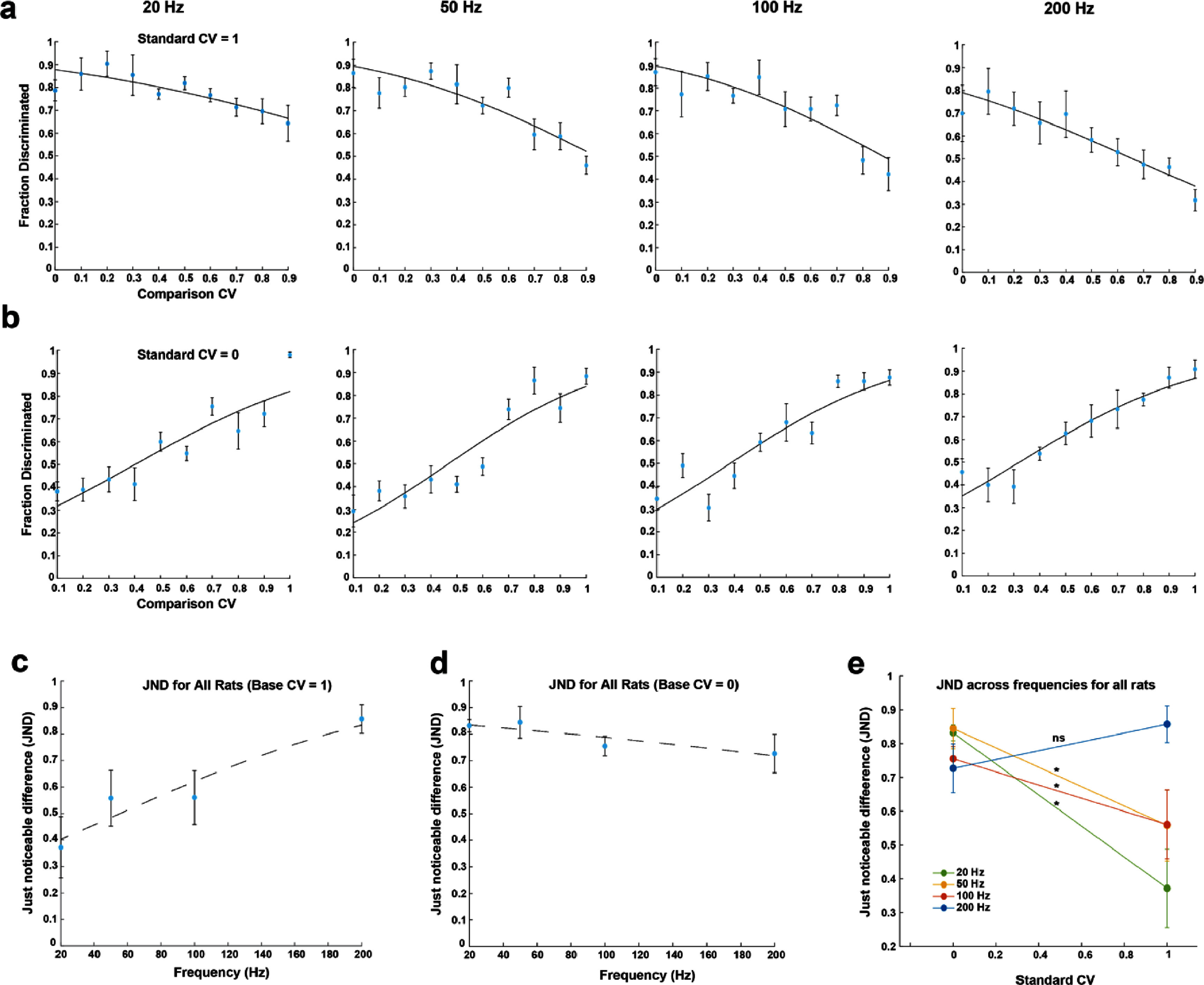
Psychometric analysis of sensory discrimination to determine just noticeable differences (JNDs) across all rats. (a) Fraction of correct trials discriminated at 20, 50, 100, and 200 Hz (*n* = 5, 5, 5, 4 rats) compared to a lower CV value (more periodic stimulus). (b) Fraction of correct trials discriminated at 20, 50, 100, and 200 Hz (*n* = 5, 5, 5, 4 rats) compared to a higher CV value (more aperiodic stimulus). (c), (d) JND as a function of frequency. (e) * indicates a statistically significant (*p* < 0.05) difference between JNDs at standard CVs of 0 and 1 based on a paired t-test. ns indicates no statistical significance. (a)–(e) Circles and error bars indicate mean ± s.e.m. Curves are sigmoid fits to the data.

Once rats learned to successfully discriminate between highly periodic (0 CV) and highly aperiodic (1 CV) stimuli, we determined JNDs by keeping standard CV of 1 while varying comparison CV from 0–0.9. Our results showed that rats were able to discriminate from a highly aperiodic stimulus at all tested frequencies (20, 50, 100, and 200 Hz), and the JNDs in CV for successful discrimination were 0.37 ± 0.12 at 20 Hz, 0.56 ± 0.11 at 50 Hz, 0.56 ± 0.10 at 100 Hz, and 0.86 ± 0.05 at 200 Hz across all rats (figures 4(a), 5(a) and (c)). To determine JNDs from a periodic stimulus we kept standard CV of 0 and varied the comparison CV from 0.1–1 for all frequencies (figure 4(b)). JNDs in CV for successful discrimination were 0.83 ± 0.02 at 20 Hz, 0.84 ± 0.06 at 50 Hz, 0.76 ± 0.04 at 100 Hz, and 0.73 ± 0.07 at 200 Hz across all rats (figures 5(b) and (d)).

Across all rats, the JNDs corresponding to the stimuli with a standard CV of 1 were significantly lower than those with a standard CV of 0 at all frequencies except 200 Hz (figure 5(e)). In addition, JNDs in CV increased as frequency increased for standard CV of 1 (figure 5(c)). In contrast, JNDs in CV decreased as frequency increased for standard CV of 0 (figure 5(d)). These results demonstrate the unique ability of rats to discriminate subtle differences in the temporal pattern of stimulation and establish that periodicity is an important parameter for generating distinct sensory perceptions.

## 4. Discussion

Building on our previous work showing that SCS can encode artificial sensory perceptions [11, 12], in this study, we investigated the ability of irregularly patterned SCS to evoke sensory perceptions by delivering pulse trains that varied in the degree of aperiodicity. Rats learned to detect and discriminate aperiodic pulse trains, which suggests that temporally-varying SCS pulse trains can be meaningfully interpreted by the brain.

**Figure 6.**
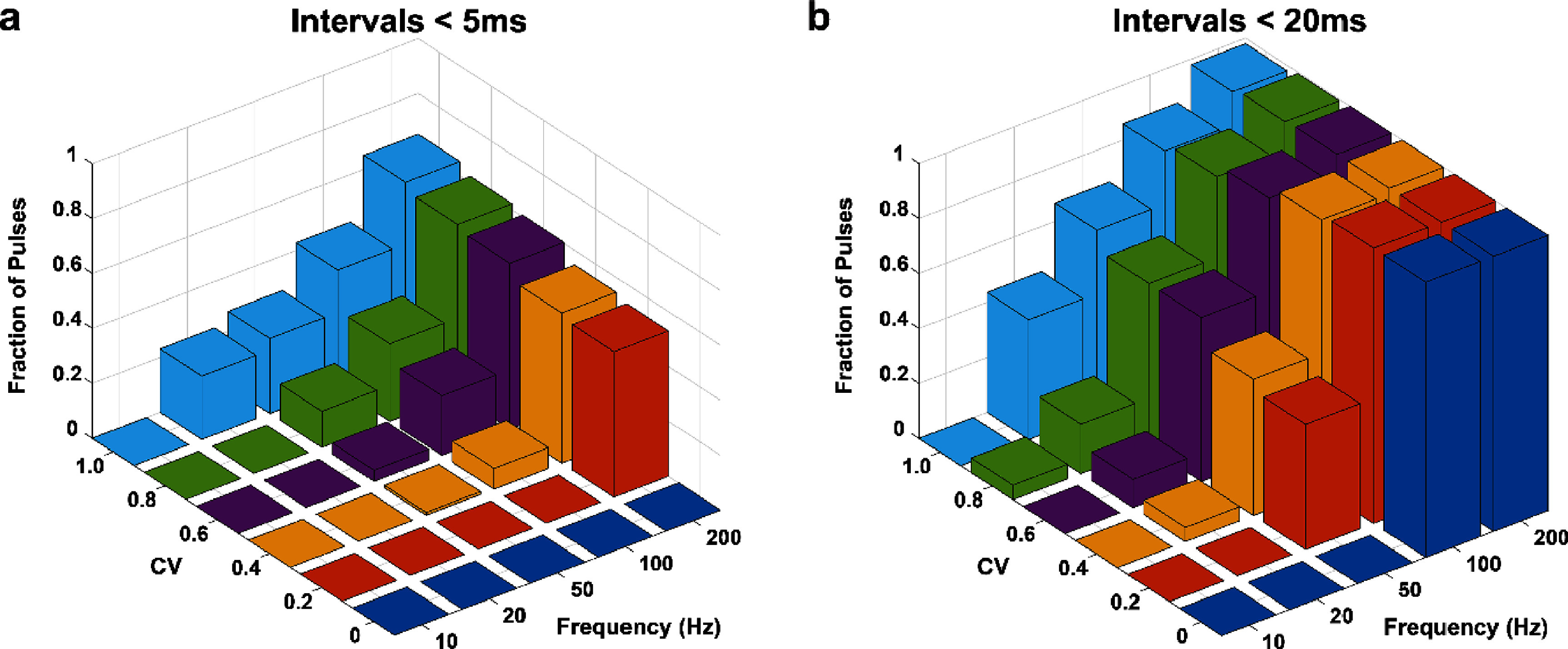
Pulse-count quantification of template patterns across CV and frequency for the two-second stimulation window. *Z*-axis displays the fraction of pulses that occurred at intervals less than (a) 5 ms (200 Hz) and (b) 20 ms (50 Hz).

Sensory detection thresholds showed a significant decreasing trend with increasing aperiodicity at 20 and 50 Hz, but not at 10, 100, or 200 Hz. This suggests that the temporal pattern of SCS is a useful parameter for modulating perceptions within certain frequency ranges and might not be applicable at all frequencies. It is also possible that certain aperiodic temporal patterns generated a higher instantaneous frequency which could facilitate sensory perception at lower amplitudes. In a previous study in which frequency and duration were simultaneously varied at a constant amplitude, rhesus monkeys could detect stimulation trains with very small durations at higher frequencies. For instance, only 2–3 pulses were required to generate a sensory percept at 200 Hz and above frequencies. In the context of our study, template patterns for 1 CV at 20 Hz and 50 Hz had approximately 23% and 27% pulses occurring at intervals less than 5 ms (200 Hz), respectively (figure 6(a)). No pulses less than 5 ms occurred for 10 Hz while only 5% pulses <20 ms were observed at 0.8 CV (figure 6(b)). This may provide an explanation for the absence of decreasing trend in overall detection thresholds as CV was varied at 10 Hz but an unexpected deviation at 0.8 CV. Based on this evidence, a significant deviation from an average low-frequency stimulus due to aperiodic patterns could explain the decreasing amplitude trend at low frequencies but not at higher frequencies.

All rats learned to discriminate between periodic (0 CV) and aperiodic stimuli (1 CV) at all frequencies. Two rats started discrimination at 50 Hz while three others started at 20 Hz (supplementary figure 1(b), supplementary figure 3) before moving on to other frequencies. JNDs in CV from aperiodic stimuli (1 CV) were consistently lower than JNDs in CV from periodic stimuli (0 CV) for all rats at all frequencies except at 200 Hz. This indicates that the difference in randomness required to discern from highly aperiodic stimuli is smaller than that required from highly periodic stimuli. It appears counterintuitive because one would assume that any slight variation from periodic stimulation would be easily discernible. However, this result might highlight that the brain can easily decode variations from a highly random pattern compared to a more regularized pattern which might be perceived as non-naturalistic at lower frequencies. These differences in JNDs were reversed when frequency discrimination was evaluated at 200 Hz (figure 5(c)), suggesting that it becomes more difficult to discriminate between aperiodic patterns at higher frequencies.

At the beginning of each discrimination session for each rat, a threshold amplitude was determined at 0 CV and kept constant throughout the session when the CVs were randomized between 0.1–1 or 0–0.9. Additionally, the number of pulses and pulse-widths were constant irrespective of the periodicity of the stimulus train. Thus, the total charge delivered was always constant across different CV values. Previous work on sensory perception using ICMS in rats suggests that the total charge delivered has the most impact on perceived intensity instead of pulse-width or current alone [25]. Even though the total charge delivered was constant in our discrimination experiments, it does not fully guarantee that the perceived intensity was the same across the two stimuli. Nevertheless, the only parameter we changed was periodicity, which altered the temporal structure of the train, while everything else remained constant. Thus, it is safe to assume that rats were discriminating the underlying temporal structure of the stimulus train. In contrast, in our previous discrimination experiments, stimulation frequency was changed using periodic pulses while keeping amplitude and pulse width constant, which resulted in different numbers of pulses delivered and, thus, different total charges delivered within the same session [12]. Altogether, our previous and current work indicates that both frequency and periodicity are important parameters to modulate the temporal structure of the train and to characterize sensory discrimination. Yet, it is not clear whether either or both determine the qualitative aspect of the reported percept. To best evaluate the qualitative aspects of perceptual differences, studies must be conducted in humans, where verbal reporting can be integrated with the psychophysical task. Nevertheless, rodents serve as important proxies to narrow down the parameter space before starting to investigate in humans.

Future experiments could explore simultaneous modulation of frequency and periodicity. Previously, it was shown that, in rodents, SCS frequency discrimination followed Weber’s law at periodic stimulation, i.e. when stimulation was delivered at 0 CV [12]. It needs to be determined whether JNDs in frequency follow Weber’s law at some or all CV values and vice versa if Weber’s law applies to JND in CV at some or all frequencies. Modulating the frequency and periodicity of SCS simultaneously can greatly expand the number of distinguishable percepts available within a fixed frequency range. But care must be taken to prevent patterns with high instantaneous frequency to avoid significantly altering the detection thresholds.

Furthermore, there are numerous additional techniques used to generate biomimetic patterns. Recent studies have shown that linear modulation of frequency, amplitude, or pulse-width is capable of altering the effect of elicited sensations [19, 26, 27]. Additionally, it is postulated that neural structures are recruited in a more naturalistic manner when these crucial parameters are tuned. The exact patterns needed to mimic the firing activity of neurons have not been characterized yet. In future, using simultaneous brain recording and SCS, we plan to investigate whether stochastic patterns or other published biomimetic patterns, such as linear frequency and amplitude modulation, evoke naturalistic neural activity. This will help evaluate the functionality of stochastic patterns in comparison to other biomimetic patterns. The dynamic range of periodicity is limited by the smallest time between consecutive pulses which is limited by the pulse-width and inter-pulse interval. Decreasing the pulse-width may provide a means to increase the dynamic range of discrimination. Although we used a gamma distribution to vary periodicity, other probability distributions, such as a lognormal distribution could be applied to generate different sets of intervals between pulses.

Lastly, the electrode location in two rats was recorded both at the beginning of the study and at the end, 4-8 months after implantation (supplementary figure 4). Additionally, the electrode impedances of nine rats were tracked for 1–6 months (supplementary table 3). There was minimal fluctuation in electrode location and impedances, demonstrating the robustness of the implantation techniques used.

In conclusion, we successfully demonstrated that the temporal pattern of SCS is an important parameter that greatly impacts the detection and discrimination of sensory perceptions. Thus, periodicity is a critical feature that needs further exploration to evoke naturalistic neural activity and generate reliable and distinguishable perceptions using SCS. Our current and previous results strongly indicate that SCS could effectively be used as a sensory neuroprosthetic technology to deliver artificial sensory feedback in clinical applications of BMIs. Finally, we envision that an SCS-based sensory neuroprosthesis will have strong implications in the sensory restoration of patients with spinal cord injuries, traumatic brain injuries, stroke, and amputations.

## Data Availability

The data cannot be made publicly available upon publication because they are not available in a format that is sufficiently accessible or reusable by other researchers. The data that support the findings of this study are available upon reasonable request from the authors.

## References

[1] Collinger J L, Wodlinger B, Downey J E, Wang W, Tyler-Kabara E C, Weber D J, McMorland A J, Velliste M, Boninger M L, Schwartz A B (2013). High-performance neuroprosthetic control by an individual with tetraplegia. Lancet.

[2] Hochberg L R (2012). Reach and grasp by people with tetraplegia using a neurally controlled robotic arm. Nature.

[3] Bouton C E (2016). Restoring cortical control of functional movement in a human with quadriplegia. Nature.

[4] Vansteensel M J (2016). Fully implanted brain-computer interface in a locked-in patient with ALS. New Engl. J. Med..

[5] Flesher S N, Downey J E, Weiss J M, Hughes C L, Herrera A J, Tyler-Kabara E C, Boninger M L, Collinger J L, Gaunt R A (2021). A brain-computer interface that evokes tactile sensations improves robotic arm control. Science.

[6] Swan B D, Gasperson L B, Krucoff M O, Grill W M, Turner D A (2018). Sensory percepts induced by microwire array and DBS microstimulation in human sensory thalamus. Brain Stimul..

[7] Armenta Salas M (2018). Proprioceptive and cutaneous sensations in humans elicited by intracortical microstimulation. elife.

[8] Raspopovic S (2014). Restoring natural sensory feedback in real-time bidirectional hand prostheses. Sci. Transl. Med..

[9] Lempka S F, Patil P G (2018). Innovations in spinal cord stimulation for pain. Curr. Opin. Biomed. Eng..

[10] Shealy C N, Mortimer J T, Reswick J B (1967). Electrical inhibition of pain by stimulation of the dorsal columns: preliminary clinical report. Anesth. Analg..

[11] Yadav A P, Li D, Nicolelis M A L (2020). A brain to spine interface for transferring artificial sensory information. Sci. Rep..

[12] Yadav A P, Li S, Krucoff M O, Lebedev M A, Abd-El-Barr M M, Nicolelis M A L (2021). Generating artificial sensations with spinal cord stimulation in primates and rodents. Brain Stimul..

[13] Chandrasekaran S, Nanivadekar A C, McKernan G, Helm E R, Boninger M L, Collinger J L, Gaunt R A, Fisher L E (2020). Sensory restoration by epidural stimulation of the lateral spinal cord in upper-limb amputees. elife.

[14] Tan D, Tyler D, Sweet J, Miller J (2016). Intensity modulation: a novel approach to percept control in spinal cord stimulation. Neuromodulation.

[15] Deer T (2018). Success using neuromodulation with BURST (SUNBURST) study: results from a prospective, randomized controlled trial using a novel burst waveform. Neuromodulation.

[16] Fishman M (2021). Twelve-Month results from multicenter, open-label, randomized controlled clinical trial comparing differential target multiplexed spinal cord stimulation and traditional spinal cord stimulation in subjects with chronic intractable back pain and leg pain. Pain Pract..

[17] Long K H, Lieber J D, Bensmaia S J (2022). Texture is encoded in precise temporal spiking patterns in primate somatosensory cortex. Nat. Commun..

[18] Panzeri S, Petersen R S, Schultz S R, Lebedev M, Diamond M E (2001). The role of spike timing in the coding of stimulus location in rat somatosensory cortex. Neuron.

[19] Formento E, D’Anna E, Gribi S, Lacour S P, Micera S (2020). A biomimetic electrical stimulation strategy to induce asynchronous stochastic neural activity. J. Neural Eng..

[20] O’Doherty J E, Lebedev M A, Li Z, Nicolelis M A (2012). Virtual active touch using randomly patterned intracortical microstimulation. IEEE Trans. Neural Syst. Rehabil. Eng..

[21] Yadav A P, Fuentes R, Zhang H, Vinholo T, Wang C-H, Freire M A M, Nicolelis M A L (2014). Chronic spinal cord electrical stimulation protects against 6-hydroxydopamine lesions. Sci. Rep..

[22] Pais-Vieira M, Yadav A P, Moreira D, Guggenmos D, Santos A, Lebedev M, Nicolelis M A L (2016). A closed loop brain-machine interface for epilepsy control using dorsal column electrical stimulation. Sci. Rep..

[23] Rees B, Borda E, Nicolelis M A, Yadav A P (2022). Closed-loop spinal cord stimulation is superior in restoring locomotion in rodent models of Parkinson’s disease. bioRxiv Preprint.

[24] Hanson T L, Omarsson B, O’Doherty J E, Peikon I D, Lebedev M A, Nicolelis M A (2012). High-side digitally current controlled biphasic bipolar microstimulator. IEEE Trans. Neural Syst. Rehabil. Eng..

[25] Bjanes D A, Moritz C T (2019). A robust encoding scheme for delivering artificial sensory information via direct brain stimulation. IEEE Trans. Neural Syst. Rehabil. Eng..

[26] Valle G (2018). Comparison of linear frequency and amplitude modulation for intraneural sensory feedback in bidirectional hand prostheses. Sci. Rep..

[27] Graczyk E L, Schiefer M A, Saal H P, Delhaye B P, Bensmaia S J, Tyler D J (2016). The neural basis of perceived intensity in natural and artificial touch. Sci. Trans. Med..

